# Single Diabetic QTL Derived from OLETF Rat Is a Sufficient Agent for Severe Diabetic Phenotype in Combination with Leptin-Signaling Deficiency

**DOI:** 10.1155/2012/858121

**Published:** 2012-12-05

**Authors:** Hiroyuki Kose, Takahisa Yamada, Kozo Matsumoto

**Affiliations:** ^1^Department of Life Science, Division of Natural Sciences, International Christian University, Mitaka, Tokyo 181-8585, Japan; ^2^Division for Animal Research Resources, Institute of Health Biosciences, The University of Tokushima Graduate School, Tokushima 770-8503, Japan; ^3^Laboratory of Animal Genetics, Graduate School of Science and Technology, Niigata University, Niigata 950-2181, Japan; ^4^Department of Animal Medical Sciences, Faculty of Life Sciences, Kyoto Sangyo University, Kyoto 603-8555, Japan

## Abstract

Obesity has been considered one of the leading causative agents for diseases such as type 2 diabetes, stroke, and heart attack. Due to their complex etiology, establishing auseful animal model is increasingly crucial for better molecular understanding of how obesity influences on disease development. OLETF rat is a spontaneous model of type 2 diabetes. We mapped 14 hyperglycemia QTLs in the genome of the OLETF rat and subsequently generated a panel of congenic strains each possessing OB-R mutation in F344 genetic background. Here we show that one of the loci, *Nidd2/of*, is highly responsive to obesity. When leptin receptor mutation is introgressed into the *Nidd2/of* congenic strain, the rat showed hyperglycemia equivalent to that of the parental OLETF rat. This suggests that the *Nidd2/of* locus has a strong genetic interaction with leptin signaling pathway. Furthermore, when another hyperglycemia *QTL Nidd1/of* is additionally combined, the strain developed overt diabetes. A single QTL dissected out in spontaneous model normally exerts only mild effect on the quantitative trait, which makes it difficult to clone the gene. Our new model may help not only to identify the causative gene but also to investigate how obesity interacts with a QTL to regulate diabetic traits.

## 1. Introduction

Diabetes is a major global public health burden in recent years. The worldwide growth of the patient populations of type 2 diabetes (T2D) is on the sharp rise and the number is predicted to double by 2030 [1, 2]. Consequently, there has been an increasing demand of mechanistic and drug development research on the disease. Like other common diseases, type 2 diabetes is polygenic and results from a complex interplay among multiple heritable as well as environmental components [3].

In humans genome-wide association study (GWAS) represents the most widely explored approach for genetic analysis of multifactorial common diseases including T2D [4]. However, genetic heterogeneity and enormous variations in exposure levels to environmental factors make it difficult to identify type 2 diabetes susceptibility loci in humans. Yet recent advancements of high performance sequencing and gene expression technologies have allowed to search for the common forms of the genetic variants which underlie “common” type 2 diabetes or obesity in human [5–7]. In spite of these efforts, there have been only a few examples in which diabetic genes have been confirmed using transgenic studies [8].

Therefore, from the prospective of complementation of the human study, it is highly crucial to approach the issue by taking advantage of appropriate experimental models [9, 10]. The OLETF rat is a widely studied spontaneous diabetes model with characteristics such as late onset (after 18 weeks of age) of hyperglycemia, mild obesity, and polygenic nature of the phenotypes [11]. Traditional genetic analyses in the OLETF have been based on mapping QTL using microsatellite markers, followed by genetic isolation of QTL in congenic strains [12–14]. Although the molecular characterization of the loci are yet to come, the careful characterization of the congenic rats have provided some useful insight into the potential mechanisms in which the causative mutations operate [15–17]. For example, it was shown that one of the hyperglycemic QTL identified in our study, *Nidd2/of,* not only causes hyperglycemia but also increases adiposity, suggesting that the QTL may make the strain more susceptible to obesity [15]. In order to address how the diabetic effect of *Nidd2/of* is influenced by the presence of severe obese condition, we combined *Nidd2/of* locus and leptin receptor mutation that is derived from the Zucker Fatty rat [18]. Furthermore, because it was also demonstrated that there is an epistatic interaction between *Nidd2/of* and *Nidd1/of*, another major hyperglycemic QTL in the genome of the OLETF rat, we set out further to examine the genetic interaction in the presence of leptin receptor mutation. 

## 2. Materials and Methods

### 2.1. Rat Strains and Animal Procedures

All rats were kept under specific pathogen-free condition. The temperature (21 ± 2°C), humidity (55 ± 10%), and air conditioning were all controlled. Rats had free access to tap water and standard laboratory chow (MF; Orietnal Yeast CO., Tokyo, Japan) and were maintained at a 12-h light and dark cycle (7 am/7 pm). Animal procedures used in this study were approved by the University of Tokushima Animal Experimentation Committee.

### 2.2. Congenic Strain Breeding

Congenic animals were constructed by the speed congenic method [14, 19]. First of all, leptin-receptor (OB-R) mutation which is derived from Zucker Fatty rats was introduced into the Fischer-344 rat (F344/Crj) background [18, 20]. The leptin receptor is mapped to q33 on the chromosome 5. The Zucker Fatty rat (Zucker/Slc) was obtained from Japan SLC, Inc. (Hamamatsu, Japan). Male Zucker Fatty and female F344 rats were crossed to yield F1 progeny. Subsequently, five generations of back-cross matings were made between male progenies and female F344 rats. At each generation, microsatellite-based genotyping was performed to select the best male that harbored OB-R mutation and the largest F344 recipient genome segment in the remaining genome region. Because homozygotes for OB-R mutation is sterile, the heterozygotes animals are selected every generation in order to maintain the strain. The strain was named F.ZF-*lepr*. Other congenic strains were named similarly.

 Once the congenic strain possessing OB-R mutation in the F344 background was established, F.ZF-lepr was crossed with F.O-*Nidd1/of*, F.O-*Nidd2/of*, F.O-*Nidd1&2/of* in order to generate corresponding double or triple congenic strains [14]. Chromosomal regions for *Nidd1/of* and *Nidd2/of* are approximately 26.6 cM defined by D7Mgh16 and D7Mgh20 on chromosome 7 and 29.5 cM defined by D14Rat23 and D14Rat12 on chromosome 14, respectively. The congenic rat that introgressed *Nidd1/of* and OB-R was named, F.ZF/O-*lepr*, *Nidd1/of*. Other congenic strains were named accordingly.

### 2.3. Genotyping

DNA isolation and polymerase chain reaction (PCR) amplification of microsatellite markers were performed as described previously [13]. The primers for microsatellite markers were purchased from Invitrogen (Carlsbad, CA). For identification of OB-R mutation, following primers were used [20].

### 2.4. OGTT Analysis

Oral glucose tolerance test (OGTT) were performed essentially the same way as described previously [13]. Briefly, male rats of 15 weeks of age were fasted overnight; blood glucose levels were measured with ADVANTAGE II (Roche) at 0, 30, 60, 90, and 120 min after oral administration of 2.0 g glucose (in a 2.8 M glucose solution) per kilogram of body weight. The serum immunoreactive insulin levels were determined at fasting with an ELISA for mouse/rat insulin (Morinaga, Japan). The serum levels of total cholesterol, triglycerides, non-esterified fatty acids were determined with reagents from Wako, Japan. Serum leptin and adiponectin were measured with ELISA kits of Morinaga (Japan) and Otsuka (Japan), respectively. One week after OGTT, fat tissues were dissected and weighed for mesenteric, retroperitoneal, and epididymal fat pad [21]. 

### 2.5. Statistical Analysis

All values are expressed as means ± SE unless stated otherwise. The statistical significance of differences was evaluated using ANOVA with a posthoc test, Scheffe's test (StatView, SAS Institute, INc.) for comparing all traits among the congenic strains.

## 3. Results

Double congenic strain showed significantly elevated glucose levels.

**Figure 1:**
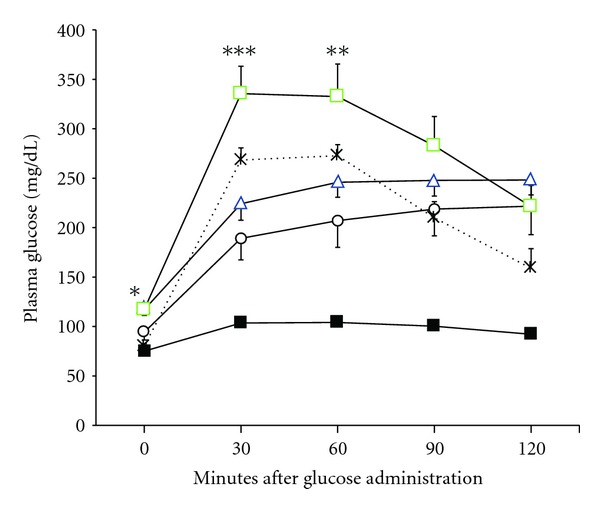
Effects of oral glucose injection on plasma glucose of the congenic strains. Glucose tolerance in 15-week-old rats of the congenic strains F.Z-*lepr* (circles), F.Z/O-*lepr*, *Nidd1/of* (triangles), F.Z/O-*lepr*,   *Nidd2/of* (squares), OLETF rats (crosses), and F344 (filled squares). *P < 0.05, **P < 0.01, ***P < 0.001 versus F.Z-*lepr*.

Since hyperglycemic QTLs were identified by intercross between OLETF and F344 rats, subsequent congenic strains were constructed by introgressing OLETF-derived QTL into F344 background [14]. Therefore, first we aimed to construct congenic strain that possesses Zucker Fatty-derived leptin receptor mutation in the background of F344 rats [18]. The resultant strain, F.ZF-*lepr*, showed mild hyperglycemia at 15 weeks of age (Figure 1). There were no significant difference in the glucose levels between the F.ZF-*lepr* and prediabetic young OLETF rat, suggesting that in the normoglycemic F344 genetic background a single mutation in the leptin receptor is not quite sufficient to develop overt diabetic phenotypes. The result is also consistent with the fact that Zucker Fatty (ZF), unlike the Zucker Diabetic Fatty (ZDF) rat, is relatively normal for the glucose metabolism [22]. Thus, without additional genetic modifications, the defected leptin signaling alone at least by this mutant allele in this genetic background is unable to cause pronounced diabetes phenotype particularly at the young age. In contrast, when *Nidd2/of* locus was added to the F.ZF-*lepr*, the glucose levels elevated significantly to higher than that of the F.ZF-*lepr* rat (Figure 1). Because the single congenic rat possessing only *Nidd2/of* locus in the genome of F344 rat showed significant yet mild hyperglycemia [14, 17], this result indicates that the effect of *Nidd2/of* is amplified by obesity and/or the deregulation of leptin signaling. This epistatic relation between the *Nidd2/of* and *lepr* is unique, because when *Nidd1/of*, another major hyperglycemic QTL and leptin mutation were combined, the glucose levels mildly increased but did not achieve statistical significance.

Next we tested the epistatic interaction between *Nidd1/of* and *Nidd2/of* when the leptin signaling is severely impaired [17]. We found that most of the rats tested on the OGTT analysis died apparently due to hyperglycemic shock, indicating that the triple congenic rat is even more severely diabetic. Consistent with this, the triple-congenic rat showed extremely high glucose levels in the fast condition (Table 1). Given that *Nidd1/of* locus alone did not exert any hyperglycemic effect, the result implies that there is a strong genetic interaction between *Nidd2/of* and *Nidd1/of* loci.

Lipid metabolisms are highly deregulated in the congenic strain.

In order to gain further insight, we compared various biochemical parameters for the fasting state (Table 1). As expected, the body weight of the F.ZF-*lepr* is significantly increased from the F344. However, both F.ZF/O-*lepr*, *Nidd2/of* and F.ZF/O-*lepr*, *Nidd1&2/of* showed rather decreased body weight in comparison to the F.ZF-*lepr* or F.ZF/O-*lepr*, *Nidd1/of*. Diabetic patients lose weight as the disease progresses. We think this is what is reflected in the body weight of the congenic rat possessing *Nidd2/of* locus. Accordingly fat weights of F.ZF/O-*lepr*, *Nidd2/of* and F.ZF/O-*lepr*, *Nidd1&2/of* also decreased compared with F.ZF-*lepr* or F.ZF/O-*lepr*, *Nidd1/of*, suggesting again the critical diabetic condition in these strains.

Total cholesterol and non-esterified fatty acids were increased in the F.ZF/O-*lepr*, *Nidd1/of* rat. Although the statistical significance was not detected, triglycerides levels were also found elevated. However, the other more diabetic strains did not show any difference in these parameters in comparison to the F.ZF-*lepr*, which is again likely due to the significant fat loss. Given that the F.ZF/O-*lepr*, *Nidd1/of* was not as hyperglycemic, the high in the lipid contents in the plasma may reflect the prediabetic condition of this strain.

Insulin levels are sharply raised in the rat with *lepr* mutation. This is presumably due to the null condition of the leptin signaling [18]. It is interesting that the addition of the Nidd QTLs tended to further aggravate the insulin resistance, indicating that consistent with our previous report, *Nidd* locus influences negative impact on the insulin action [17].

## 4. Discussion

In this study we showed that the single QTL, namely *Nidd2/of,* is sufficient to induce severe hyperglycemia if the leptin signaling is simultaneously suppressed. It is important that as demonstrated previously, the *Nidd2/of* by itself showed only mild hyperglycemia just like other hyperglycemic QTLs [14]. This implies that there are some strong genetic interactions between the components of leptin signaling and/or resultant obese physiology and causative genes mapped to the QTL.

 In mouse as well, whether single loss-of-function mutation in either leptin or its receptor develops severe diabetes depends on the genetic background. For example, BTBR T(+) (BTBR) mouse develops overt diabetic symptoms whereas C57BL/6(B6) strain shows only mild hyperglycemia using the *Lep* ^*ob*^ mutation as a stressor [23, 24]. Similarly the Zucker Diabetic Fatty (ZDF) rat, the special subline of Zucker fatty strain, shows diabetic phenotypes presumably due to diabetes-susceptibility alleles [25, 26]. Therefore, although leptin signaling is highly crucial for understanding the etiology of the diabetes, genetic elements that place demand on insulin action in response to obese states should be a focus of research using diabetes animal models. Indeed, the strategy of sensitized screens whereby a severe stressor provokes abnormal metabolic phenotypes that would be otherwise near silent started bearing fruits and led to identification of the causative genes recently [27–30].

It is noteworthy that the *Nidd1/of* is quite a contrast to the *Nidd2/of* in that it did not contribute to any further hyperglycemia in the condition of leptin signaling deficiency. Because the *Nidd1/of *alone is sufficient to influence glucose metabolism in the F344 genetic background, the strong influence of leptin receptor mutation may be “masking” the effects of the QTL. In this respect, it deserves the attention that the epistasis is obvious in the triple congenic strain, which leads us to speculate that the two QTLs play a major role for the expression of the diabetes as the animal becomes obese due primarily to the influences of other genetic components.

One of the characteristics of the OLETF rat is that in this strain the diabetic symptoms become overt after 20 weeks of age or older [31]. In the current study the age of the rats that were analyzed is 15 weeks old. Therefore, the hyperglycemic phenotype of OLETF rat is not fully developed. Even so it is striking that the glucose levels of the *Nidd2/of*-possessing congenic strains are much higher than that of the parental OLETF strain. This suggests that there may exist loci that suppress the diabetic QTLs in the genome of the OLETF rat, which may not be too surprising considering that the expression of the complex traits is the result of intricate interplay among numerous genetic components, which is the case for the hypertension model [32].

In conclusion, the new congenic strain carrying single QTL derived from the OLETF rat shows severe hyperglycemia when combined with obese phenotype via leptin receptor mutation. The hyperglycemia was further aggravated by the addition of yet another QTL, implying the strong epistasis between them. It is hoped that this model will help to shed a new light on the molecular mechanisms of type 2 diabetes in humans.

## Figures and Tables

**Table 1:** Comparison of metabolic parameters.

	F344	F.ZF-*lepr *	F.ZF/O-*lepr, Nidd1/of *	F.ZF/O-*lepr, Nidd2/of *	F.ZF/O-*lepr, Nidd1* and *2/of *	OLETF
	(n = 8)	(n = 14)	(n = 19)	(n = 11)	(n = 4)	(n = 5)
Glucose (mg/dL)	75.1 ± 2.6	94.5 ± 4.9	117.2 ± 6.1	117.4 ± 5.1	172.7 ± 11.2***	80.2 ± 2.6
Insulin (ng/mL)	2.21 ± 0.22	48.8 ± 5.2	78.4 ± 9.3*	70.9 ± 11.9	72.0 ± 10.5	1.63 ± 0.3*
TCHO (mg/dL)	64.8 ± 2.4	234.2 ± 26.2	439.3 ± 38.4**	227.2 ± 19.0	370.5 ± 55.9	97.9 ± 3.7
TG (mg/dL)	174.2 ± 15.0	1170 ± 165	1720 ± 145	777 ± 106	617 ± 122	122.7 ± 14.3**
NEFA (mEq/L)	0.87 ± 0.13	4.28 ± 0.50	6.33 ± 0.5*	3.45 ± 0.4	2.11 ± 0.4	0.75 ± 0.05*
Fat weight (g)						
Mesenteric fat	7.1 ± 0.4	15.6 ± 0.8	14.5 ± 0.4	10.4 ± 0.5***	11.4 ± 1.7*	9.5 ± 0.5***
Retroperitoneal fat	7.6 ± 0.4	15.0 ± 0.8	14.5 ± 0.4	13.3 ± 0.6	13.2 ± 1.0	15.5 ± 1.2
Epididymal fat	9.0 ± 0.5	13.8 ± 0.6	12.5 ± 0.4	9.9 ± 0.3***	9.2 ± 1.0***	8.6 ± 0.5***
Adiposity index (%)^a^						
Mesentric fat	2.23 ± 0.11	3.63 ± 0.16	3.52 ± 0.06	2.95 ± 0.18*	3.23 ± 0.34	1.96 ± 0.07***
Retroperitoneal fat	2.39 ± 0.09	3.47 ± 0.13	3.52 ± 0.07	3.72 ± 0.86	3.79 ± 0.11	3.20 ± 0.19
Epididymal fat	2.81 ± 0.11	3.23 ± 0.13	3.02 ± 0.06	2.79 ± 0.11	2.62 ± 0.14	1.79 ± 0.07*
Body weight (30 w) (g)	314.3 ± 5.6	417.5 ± 8.5	403.5 ± 9.3	349.8 ± 7.9***	343.8 ± 13.6**	481.0 ± 13.3*

TC: total cholesterol; TG: triglycerides; NEFA: nonesterified fatty acids.

Sixteen-week-old fasted males were used for all measurements except glucose, insulin, and body weight, which were measured during OGTT analysis at 15 weeks of age.

Data are shown as means ± SE.

^a^Adipocity index was determined using each fat pad and body weight (percentage of fat weight/body weight).

*P < 0.05; **P < 0.01; ***P < 0.001 versus F.ZF-*lepr*.

F344 data are shown as reference, thus no statistical analysis was performed.
